# Hereditary Thoracic Aortic Diseases

**DOI:** 10.3390/diagnostics14010112

**Published:** 2024-01-04

**Authors:** Gaia Spaziani, Francesca Chiara Surace, Francesca Girolami, Francesco Bianco, Valentina Bucciarelli, Francesca Bonanni, Elena Bennati, Luigi Arcieri, Silvia Favilli

**Affiliations:** 1 Pediatric and Transition Cardiology, Meyer Children’s Hospital IRCCS, 50139 Florence, Italy; francesca.girolami@meyer.it (F.G.); elena.bennati@meyer.it (E.B.); silvia.favilli@meyer.it (S.F.); 2 Cardiovascular Sciences Department, AOU “Ospedali Riuniti”, 60126 Ancona, Italy; francescachiara.surace@ospedaliriuniti.marche.it (F.C.S.); francesco.bianco@ospedaliriuniti.marche.it (F.B.); valentina.bucciarelli@ospedaliriuniti.marche.it (V.B.); luigi.arcieri@ospedaliriuniti.marche.it (L.A.); 3 Department of Experimental and Clinical Medicine, School of Cardiology, Faculty of Medicine, University of Study of Florence, 50121 Florence, Italy; francesca.bonanni@unifi.it

**Keywords:** hereditary aortopathies, thoracic aortic disease, genetics, aortic dilation, multimodality imaging

## Abstract

Advances in both imaging techniques and genetics have led to the recognition of a wide variety of aortic anomalies that can be grouped under the term ‘hereditary thoracic aortic diseases’. The present review aims to summarize this very heterogeneous population’s clinical, genetic, and imaging characteristics and to discuss the implications of the diagnosis for clinical counselling (on sports activity or pregnancy), medical therapies and surgical management.

## 1. Introduction

Hereditary thoracic aortic diseases (HTADs) are a heterogeneous group of rare pathologic conditions related to/caused by a large variety of genetic errors, potentially associated with severe complications in some patients (aortic dilatation, dissection or tortuosity) and requiring an appropriate management strategy, from medical follow-up to pharmacologic treatment and timely aortic surgery.

A section dedicated to thoracic aortopathies has been included in the most recent guidelines of the European Society of Cardiology concerning adult congenital heart disease [1]. The ‘model’ disease of HTADs was considered to be Marfan syndrome (MS), but aortic diseases associated with bicuspid aortic valve (BAV) were also included. Clinical overlap between different HTADs has been described, requiring caution in establishing the diagnosis. Even if the progression of aortic dilation is an essential prognostic indication in all HTADs, defining the underlying pathologic condition is crucial to predicting the risk of complications [1].

Identifying the genetic error may give important information concerning the risks and prognosis, eventually allowing gene-targeted medical strategies [2]. However, several causative genes remain unknown [2,3].

Extracardiac features are often recognized in HTADs; therefore, a multidisciplinary approach should be planned, and a dedicated ‘Aortopathy Clinic’ has been advocated [4,5].

We report a review of the literature and narrative synthesis of HTADs, from clinical presentations to genetic features, from diagnostics tools to medical therapies and surgical management. Original articles, observational studies and international guidelines were identified from the PubMed database. The search words included a disease term of HTADs such as Marfan syndrome, Loeys–Dietz syndrome, Turner syndrome, vascular Ehlers–Danlos syndrome, osteogenesis imperfecta, Shprintzen–Goldberg syndrome and bicuspid aortic valve.

## 2. Clinical Conditions

The detection of aortic dilatation, especially in younger patients, can underlie different clinical conditions with different prognoses and therapeutic indications [6]. Therefore, a definite diagnosis is paramount and affects whether a more or less aggressive therapeutic approach is used. Clinical assessment is the ‘starting point’ of the diagnostic process, but further investigations, comprising genetics, are usually required to define the diagnosis [7].

Marfan syndrome (MS) is the prototype of syndromic HTADs [1]; cardiovascular abnormalities (dilation of the aorta) and extracardiac features (skeletal abnormalities, ectopia lentis, dural ectasia) are essential for clinical diagnosis. Survival was significantly reduced in the previous cohorts of patients with MS, with death usually occurring between the third and the fifth decades of life. Early diagnosis and timely surgery have drastically reduced mortality, leading to near-normal life expectancy [8]. The diagnosis is based on clinical criteria and a scoring system, of which an aortic root aneurysm is a major determinant; Ghent criteria were revised in 2010 with greater emphasis on the role of genetic testing [9]. Among cardiovascular manifestations, aortic root aneurysms and dissection are the most characteristic and are the main determinants of prognosis. The dissection risk is related to the aortic root dimensions and significantly increases when the diameter exceeds 5 cm. However, additional risk factors for aortic rupture are represented by the aortic growth rate, family history of dissection, and hypertension [1]. Pregnancy further increases the risk of dissection (see paragraph). The probability of aortic dissection rises with age and is exceedingly low in pediatric patients.

Vascular lesions, including lesions of the aorta, are typical features in another rare severe connective disorder, Loeys–Dietz syndrome (LDS). Different clinical characteristics and prognoses have been described depending on the type of genetic mutation (see paragraph). While aortic dissection has been described in all subgroups, acute aortic complications can occur at smaller aortic diameters in patients with a mutation in TGFBR2 (type 2); higher rates of annual aortic root size increase were described in the same subgroup, even in pediatric age [10]. According to the literature, the rate of aortic aneurysms is related to the genetic mutation. In general, the dissection risk is higher in LDS when compared with other HTADs and requires great caution, especially during pregnancy [10].

Congenital cardiovascular abnormalities, including BAV, aortic coarctation (AC) and aortic dilation (most commonly at the sinotubular junction and the ascending aorta) are reported in approximately 50% of women with TS. Women with TS usually have a short stature, so aortic diameters should always be indexed for body surface area. Notably, dissection may occur at the ascending aorta and the descending aorta [11]. The risk of aortic dissection has been related to BAV and/or AC and aortic dilatation. However, this population’s frequency of aortic dissection still needs to be defined [12,13]. Thus, a proper transition from pediatric to adult care and a long-life cardiologic follow-up has been recommended for patients with TS.

Cardiac involvement and aortic dilation are rare in ‘classical’ and hypermobile Ehlers–Danlos syndrome (EDS), the most common connective tissue disorder [14]. Therefore, without a family history of aortic disease/complications, a regular clinical and echocardiographic follow-up should not be planned for these patients. On the contrary, close controls are required for vascular EDS (vEDS or EDS IV), which is a rare condition, accounting for less than 5% of cases of EDS, resulting from a mutation in the gene coding for type III collagen. Aortic complications are frequent and severe in patients with vEDS, and it is not uncommon that the first diagnosis occurs due to an aortic rupture. Clinical suspicion, crucial to select patients for genetic screening, has a good sensibility (around 90%) but a low sensitivity. Patients with EDS IV usually have thin and fragile skin with visible veins, characteristic facial features and easy bruising, joint hyperextensibility and skin hypermobility, which are less common in other EDS forms [15].

Osteogenesis imperfecta (OI) is a connective tissue disorder characterized by bone fragility, with many perinatal fractures, severe bowing of long bones, decreased mineralization, and death in the perinatal period due to respiratory insufficiency [16]. OI patients are at increased risk of cardiovascular disease of variable severity. Aortic aneurysm/dissection is one of the rarer but potentially serious cardiovascular complications of OI [17].

The Shprintzen–Goldberg syndrome (SGS) is characterized by craniosynostosis, a marfanoid pattern, and anomalies in skeletal, neurological, cardiovascular, and connective tissue. Arachnodactyly, pectus deformities, camptodactyly, scoliosis, and joint hypermobility are all common skeletal manifestations [18]. The majority of craniofacial, skeletal, skin, and cardiovascular manifestations of MS and LDS are represented by SGS. Aortic root dilatation is less frequent in SGS than in LDS or MS, but when present in individuals with SGS, it can be severe [19].

**Figure 1:**
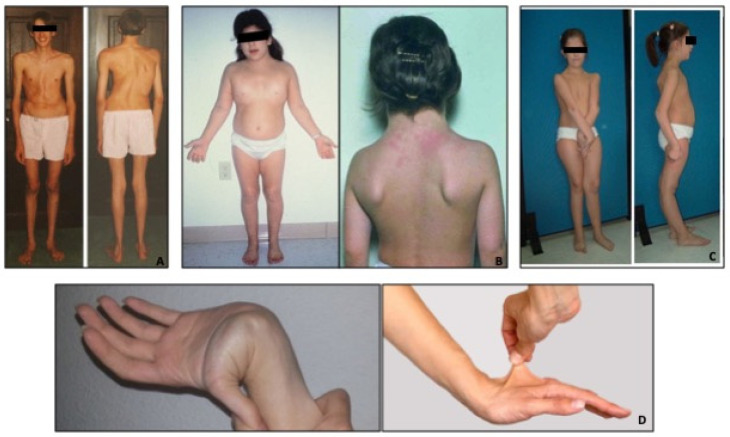
Phenotypes of the most common genetic syndromes associated with aortic dilation. (Panel **A**): Marfan syndrome characterized by long bone overgrowth, arachnodactyly scoliosis, pectus deformities, and tall stature. (Panel **B**): Turner syndrome characterized by short stature, webbed neck, broad chest and widely spaced nipples, and low hairline. (Panel **C**): Loyes–Dietz syndrome characterized by hypertelorism, scoliosis, bifid uvula, club feet, loose joints, and longer fingers. (Panel **D**): Vascular Ehlers–Danlos syndrome characterized by small joint hypermobility and thin, translucent skin.

The phenotypes of the most common syndromes are represented in Figure 1.

In the presence of an aortic dilation, additional cardiac and extracardiac features may address the etiologic diagnosis (see Table 1).

## 3. Genetics in HTADs

HTAD can present as an isolated finding or as part of a genetic syndrome [20]. Advances in technology for DNA sequencing have identified several causative genes for HTAD, including genes responsible for MS, LDS and EDS IV [9]. They are inherited autosomal dominant disorders with incomplete penetrance of connective tissue and with an inheritance risk of 50%, caused by genes involved in the TGFß pathway and genes associated with components of the extracellular matrix [2]. Clinical assessment is the ‘starting point’ of the diagnostic process, but further investigations, comprising genetics, are usually required to define the diagnosis [7]. Genetic testing can aid in the diagnosis and be useful for medical therapy, surveillance and planning the sports activity restrictions necessary, especially for pediatric patients; additionally, identifying the underlying gene triggering HTAD provides vital information to identify family members at risk for the disease [7]. The most recent guidelines of the American Heart Association (AHA)/American College of Cardiology (ACC) for the diagnosis and management of aortic disease suggested performing clinical genetic testing as an integral part of the diagnostic workflow for patients with HTAD who have clinical indicators suggestive of an underlying single gene disorder [5]. The ClinGen Aortopathy Working Group have curated a list of 11 causative genes in which pathogenic variation predisposes to HTAD (FBN1, TGFBR1, TGFBR2, SMAD3, TGFB2, COL3A1, ACTA2, MYH11, MYLK, LOX, and PRKG1). A definitive or strong association with HTAD is firmly established for these genes. These panels also include genes that increase the risk of HTAD and lead to systemic features that overlap with MS, LDS, or vEDS [5]. In addition, the Working Group have curated a list of eight genes to be associated with moderate or limited HTAD (EFEMP2, ELN, FBN2, FLNA, NOTCH1, SLC2A10, SMAD4 and SKI); these potentially diagnostic genes may allow for a diagnosis of the cause of thoracic aortic enlargement but are primarily associated with other clinical features and do not carry significant risks of progression to aortic dissection.

Finally, regarding seven genes recently reported as associated with HTAD (BGN, FOXE3, HCN4, MAT2A, MFAP5, SMAD2, and TGFB3), there is currently insufficient evidence to support a definitive association, as they are novel genes, and only one or two supporting publications have been published [21]. For these reasons, the ClinGen Aortopathy Working Group have recommended screening the 11 gene panels for diagnostic purposes. The estimated mutation detection of this multigene panel ranges from 15% to 20% and is dependent on clinical selection criteria. FBN1, TGFBR1, TGFBR2, SMAD3, and TGFB2 mutations have been identified in approximately 6% to 8% of HTAD families whose members do not have syndromic features of MS or LDS. ACTA2, MYH11, MYLK, LOX, and PRKG1 mutations have been confirmed to cause HTAD without significant features of MS or LDS [22].

For these two additional gene categories, some additional rare syndromes are described (see Table 2) [22]. These genes are a heterogeneous group of genes for which the evidence is often difficult to assess. The presentation of aortic dilatation is often lacking. For example, SKI and FLNA genes are associated with syndromes in which the presentation is usually dominated by systemic features other than aortic diseases. The SGS is caused by a heterozygous mutation in the SKI gene on chromosome 1p36. There is considerable phenotypic overlap between SGS, MS and LDS [18,19].

X-linked periventricular heterotopias (PVHN) is caused by a mutation in the gene encoding filamin A (FLNA) on chromosome Xq28. Neuronal migration disorders, such as periventricular nodular heterotopia, occur when neurons do not migrate appropriately from the ventricular zone to the cortex during development, resulting in the formation of nodular brain tissue lining the ventricles. Other characteristics involve flaws in the cardiovascular system, like patent ductus arteriosus, BAV, and dilation of the sinuses of Valsalva or the thoracic aorta. Several patients with PVNH and mutations in the FLNA gene have been reported with a spectrum of connective tissue abnormalities characterized by combinations of vascular, cardiac, cutaneous, and joint-related symptoms [23].

Genetic testing should be initiated in someone with aortic diseases and performed with appropriate pre- and post-test counselling. Genetic counselling helps explain to patients and families the genetic risk and how it is inherited, assessing the family history to determine HTAD risk, assisting in cascading genetic testing and imaging for HTAD in family members, and offering psychosocial and ethical guidance. An additional benefit of panel testing is that more than one variant could contribute to disease presentation. Once a pathogenic/likely pathogenic variant is identified, family members can be tested for the variant to determine who needs ongoing vascular cardiological follow-ups [9]. In the case of identifying variants of unknown significance (VUS), this is a clinically inconclusive result that does not confirm the HTAD and therefore should not be used to identify which family members are at risk or to guide clinical management. In addition, identifying a pathogenic variant in a patient with HTAD enables prenatal and preimplantation genetic diagnoses, and specific recommendations for imaging and surgical and pharmacological treatment based on the genetic mutation are emerging [24].

### 3.1. Marfan Syndrome

MS is caused, in more than 90% of cases, by a heterozygous mutation in the FBN1 gene coding the extracellular matrix protein fibrillin-1. MS is an autosomal dominant disorder with incomplete penetrance and variable expressivity. The FBN1 is located on chromosome 1 in 5q21.1, and about 75% of patients affected by MS have a positive family history [20]. The mechanism for the gene–disease relationship is the loss of function of the protein, as a mutation in the FBN1 protein, fibrillin, results in the inability of the protein to be excreted from cells to help in the formation and stabilization of connective tissue [25]. Although there are no obvious mutational hotspots and no precise molecular mechanisms, the relationships between the location of FBN1 mutations and the phenotype have been extensively reported. For example, in patients with ectopia lentis, there is a higher probability of a missense variant affecting a cysteine residue in the first 16 exons of the gene. In contrast, mutations in the middle part of FBN1 (exons 25 to 33) are recognized as associated with neonatal MS, which is characterized by severe mitral and/or tricuspid valvular insufficiency and pulmonary emphysema. In addition, FBN1 truncating variants (nonsense, splicing and frameshift variants) are associated with an increased risk of aortic events [22].

### 3.2. Loeys–Dietz Syndrome

LDS is caused by mutations in genes encoding the components of the TGFß signaling pathway, including TGFBR1, TGFBR2, SMAD3 and TGFB2 [21]. LDS type 1 and type 2 are the two most severe and common types due to mutations in the TGFBR1 and TGFBR2 genes, respectively. Pathogenic variants in TGFBR1 and TGFBR2 are mostly missense and detected within the serine/threonine kinase. The mechanism for the gene–disease relationship is a perturbation of the TGF beta signaling pathway; however, the exact molecular mechanism remains unclear and could include the gain of function and a dominant negative [3,26]. The majority of the mutations result in aberrant kinase activity of TGFBR1.

### 3.3. Ehlers–Danlos Syndrome

According to Villefranche nosology, the different EDS types can be classified into six main subtypes. Genetic testing results are very important for sub-type classification [20]. vEDS results from pathogenic variants in the COL3A1 gene coding for type III collagen. Specific mutations in COL3A1 have been shown to have the effect of haploinsufficiency. More than 700 COL3A1 pathogenic variants have been reported, and about 50% of cases are sporadic; genotype–phenotype correlations have also been analyzed. Variants in splice site domains or frameshift variants, together with missense variants in the central triple helix collagenous domain characterized by the repeated amino acid motif Gly-X-Y, are correlated to a high risk of arterial complications [22]. Individuals with COL3A1 pathogenic variants can present with aortic dissections with minimal syndromic features [21].

### 3.4. Turner Syndrome

TS is a complex genetic disorder affecting 1/2500 live-born girls and is caused by a complete or partial absence of the X chromosome. The genetic diagnosis of TS is based on the karyotyping, and the two main groups can be identified as follows: women with 45, X and women with mosaicism. More than half of TS patients are mosaic, including one third who harbor structural X chromosome variants, including partial deletions, isochromosomes and ring chromosomes. As you might expect, the phenotypes and outcomes of mosaic TS patients tend to be milder than patients who are constitutionally 45, X in peripheral blood. For example, the frequency and severity of cardiovascular defects are significantly lower in mosaic patients [27]. In general, mosaicism modulates phenotypes by decreasing their penetrance. Somatic mosaicism is a major modifier of TS features [13].

## 4. Imaging in HTADs

In the era of multimodality imaging, diagnosis, management, and longitudinal follow-up using advanced techniques have revealed progressive dilatation of the distal aorta after aortic root replacement in MS and HTADs, as demonstrated by Lenz and colleagues [28].

In this context, echocardiography is still the primary test for the anatomical and functional assessment of the cardiac structure and its principal vessels. Echo is extremely useful in the context of the screening and follow-up of HTADs [29,30,31].

**Figure 2:**
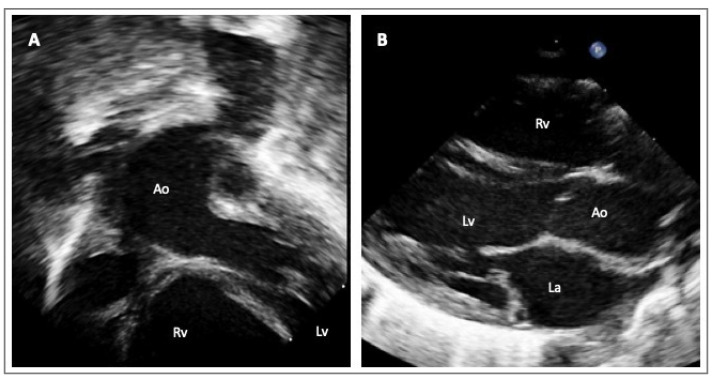
Echocardiographic images of a one-year-old boy with suspected LDS/MS presenting with an isolated dilatation of the ascending aorta. Subcostal view of the aorta (Panel **A**); parasternal long axis view (Panel **B**). Legend: left ventricle (Lv); right ventricle (Rv); aorta (Ao); left atrium (La).

In everyday clinical practice, transthoracic echocardiography (TTE) is utilized to assess the proximal aortic dimensions via the TTE parasternal long axis view. In children, or when the acoustic windows are particularly suitable in adults, it is possible to visualize more aortic segments, i.e., the mid-distal ascending aorta, moving the transducer upper to the intercostal spaces, or in apical three-chambers and five-chambers views, and mainly in children, in the modified subcostal views. The aortic arch and the proximal descending aorta measurements can be achieved via the suprasternal window in adults and children (Figure 2) [32].

**Figure 3:**
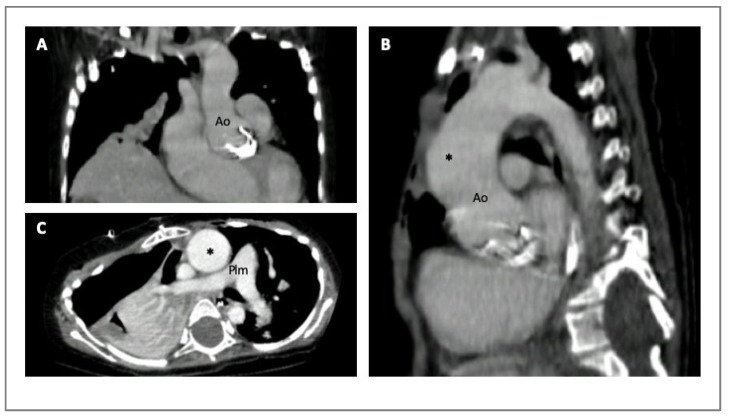
CCT images of four-year-old girl with MS that underwent previous aortic valve replacement due to severe aortic regurgitation. Coronal section (Panel **A**); sagittal section (Panel **B**); axial section (Panel **C**). The asterisk indicates the dilated ascending aorta. Legend: aorta (Ao); pulmonary artery (Plm).

Cardiac computed tomography (CCT) is a fundamental instrument in all types of aortopathies. In particular, CCT contributes to the diagnosis, risk stratification, and surgical planning at the expense of ionizing radiation exposure. Its extensive availability, along with good reproducibility and short times of acquisition, and the possibility it has to provide simultaneous luminal and mural information of the entire aorta at every level (valve, arch, ascending and descending portions) have led to the widespread utilization of computed tomography (CT) (Figure 3) [5,29,32].

**Figure 4:**
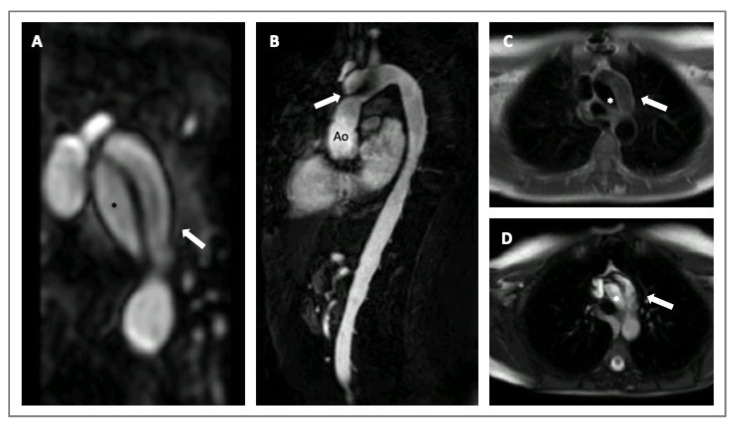
CMR imaging of a 30-years old woman with MS presenting with an acute aortic dissection diagnosed during a routine exam. Angio-MRI with post contrast-enhanced sequence (Panel **A**); cine-SSFP sagittal and axial images (Panels **B**,**D**); T1-weighted axial images (Panel **C**). The asterisks indicate the true lumen and the arrows the false lumen. Legends: aorta (Ao); steady-state free precession (SSFP).

Cardiovascular magnetic resonance (CMR) is a valid imaging tool for aorta visualization that recently demonstrated good and consistent reproducibility in various contexts. CMR can acquire multiplanar and 3D cardiac and aortic images without using iodinate contrast agents and ionizing radiation at the expense of longer scan times. Furthermore, CMR can be considered the ultimate imaging technique for follow-up comparative studies, particularly on the young [32,33]. CMR also allows for evaluating aorta biomechanical parameters, such as stiffness, distensibility, and strain, used to assess aorta elasticity in patients with MS (Figure 4) [29,32]. Recently, 3D-cine (time-resolved) phase-contrast CMR with three-directional velocity encoding (4D flow) has been developed to study intravascular flow. It quantifies flow similarly to 2D-cine phase-contrast CMR, has good scan repeatability, and allows for analyzing wall shear stress or turbulent kinetic energy in patients with HTADs [29]. In fact, van Andel and colleagues recently demonstrated that abnormal aortic hemodynamics are associated with aortic complications in patients with MS, especially if patients are male and have a haplo-insufficient type of FBN1 mutation [34].

## 5. Medical Treatment in HTADs

Medical therapy for individuals with HTAD has been conducted since 1971 when Halpern and colleagues proposed using pharmacological treatments with beta-adrenergic blockade to reduce the risk of aortic dissection in subjects with MS. Since then, hypertension management has remained a central factor and a therapeutic target for all physicians caring for adult patients with either syndromic or non-syndromic thoracic aortic disease [35].

Initially, traditional therapies for inherited thoracic acropathies were based on using beta-blockers (BBs), reducing aortic wall stress by lowering blood pressure and systemic vascular resistance, consequently reducing the progression of aortic dilatation. Then, studies on a mouse model and later on humans suggested that angiotensin receptor blockers (ARBs) treatments were potentially beneficial in vascular tissue remodeling and in reducing hemodynamic wall stress.

Most of the trials presented in the literature on HTAD pharmacological treatments concern subjects with connective tissue disorders (Table S1) [3,36,37].

The optimal medical treatment of patients with aortic aneurysms is obtained only after attaining an appropriate diagnostic setting and adequate recognition of a genetic substrate. Vascular management includes the use of pharmacological and non-pharmacological approaches, characterized as blood pressure-lowering medication, the avoidance of medications acting as stimulants or vasoconstrictors, and exercise restrictions [38].

Recognition of the type of aortopathy is crucial for subsequent management and tailored treatments. It is known that BAV is an independent risk factor for developing a progressive aortopathy. In patients with bicuspid aortopathy, cardiovascular risk factors, such as smoking and hypertension, require attention, and the AHA/ACC guidelines recommend using antihypertensive drugs such as BBs, angiotensin-converting enzyme inhibitors (ACEi), and ARBs [38].

Furthermore, specific clinical conditions, such as TS, could cause the risk of dissection at relatively small aortic diameters. Indeed, results from clinical trials suggest that the aortopathy in TS has a multifactorial origin related to genetic factors, the influence of estrogenic deficiency, and the predisposition of cardiovascular risk factors, including diabetes and hypertension; for this reason, regarding TS, all these factors must be monitored and treated early [38,39].

There are no randomized trials concerning the impact of medical therapy in reducing aortic growth or the aortic dissection risk in patients with LDS. In mouse models of LDS, the treatment with ARBs prevents aortic root dilation and associated vascular histological modifications [40]. Current guidelines recommend a lifestyle modification, regular multidisciplinary follow-ups with multi-imaging surveillance, and surgical intervention only when indicated. It seems reasonable to adopt a similar approach used for Marfan patients, with the use of ARBs, ACEi and BBs in adult and young patients, to lessen hemodynamic stress on the aorta [38,41].

If surgical treatment is indicated, the pre-operative medical management of patients with HTAD should be evaluated case by case. Although cases of deep hypotension during general anesthesia are described when ARBs or BBs are administered before surgery, medical therapy should be continued, and especially BBs should not be discontinued before surgery.

### 5.1. β-Blockers

BBs represent the first category of drugs adopted by adult patients with an MS diagnosis and is a medical standard of care for MS. Clinicians often prescribe BBs as the first-line treatment to slow the rate of aortic dilatation, which is usually observed in patients with MS [42,43].

Clinical data regarding the use of BBs in pediatric patients with syndromic or non-syndromic aortopathy are more limited regarding the use of BBs in adults. Some authors suggest a target heart rate of 60–70 bpm in adults and young patients, with a reduction of 20–30% of the maximum frequency during exercise and maximum heart rate values of 100 bpm during submaximal exercise in adults/young adults and 110 bpm in children [44]. The most widely used BBs in children are atenolol and propranolol. For individuals with MS, the early use of medical therapy independently according to the aortic diameter is reasonable, and it should be initiated early, before puberty. In fact, aortic root dilatation in patients with MS reaches its peak between 6 and 14 years of age.

In vEDS, BBs are often prescribed, with some physicians choosing alternative BBs with vasodilatory properties. In vEDS, treatment with a selective B1 blocker, celiprolol, was associated, in a study, with a reduced event rate. However, there was a small increase in brachial blood pressure, suggesting that blood pressure was not the mediator of aortic risk in these patients [45].

### 5.2. Angiotensin II Type I Receptor Blockers (ARBs)

The renin–angiotensin system plays a crucial role in the homeostasis of the cardiovascular system and represents a promising means to slow down the progression of aortic dilatation in HTAD. Clinical studies on the use of ACEi in non-atherosclerotic and hereditary aortopathies are limited, but in recent years, there has been a growing interest in it. In a small observational study of children with severe MS treated with losartan, all cases showed at least some reduction in aortic root growth compared with their progression prior to medical therapy; however, the degree of response was variable [42].

No studies are showing the benefits of ARBs in vEDS or in the case of patients with LDS. In these patients, current guidelines recommend medical blood pressure control with ARBs (especially losartan), BBs, or ACE-I to lessen hemodynamic stress on the aorta in subjects with severely progressive vascular disease. Prophylactic medication with ARB should be considered for individuals with LDS without aortic enlargement, whether they have a family history of LDS with aortic enlargement or whether the same mutation has been previously seen with vascular disease [46].

### 5.3. Other Drugs

The pharmacological inhibition of the complex mechanisms of extracellular matrix degradation or of the molecular mechanisms responsible for aortic wall destruction may be a key mechanism underlying the effects on aortic destruction, dissection, and rupture in MS. Several studies conducted on MS mouse models have demonstrated that the inhibition of metalloproteinase (MMP)-2 and -9 may be a potential strategy to ameliorate aortic dilation in MS [47]. For example, the use of doxycycline, a tetracycline-class antibiotic, proved to delay aneurysm rupture and to attenuate the aortic root grown in a model of MS. In fact, doxycycline alone or in combination with traditional drugs (BBs and ARBs) improves elastic fiber integrity, normalizes aortic stiffness and improves wall and vessel integrity [47,48]. Indeed, prolonged administration of doxycycline, at a subantimicrobial dose, effectively inhibits the function of MMP-2 and -9, thus promoting the stabilization of abdominal aortic aneurysm models (3) by preserving the elastic fiber integrity and the aortic mechanical properties through the attenuation of TGF-Beta/Smad2 signaling [48,49].

There are limited data on the efficacy of calcium channel blockers on MS, for example, concerning the efficacy of non-dihydropyridine CCB (verapamil) compared with ACEi or BB effects on central aortic pressure, conduit arterial stiffness and left ventricular function [50]. Usually, calcium channel antagonists should be used with caution when treating patients with syndromic inherited thoracic aortopathy or congenital heart disease [51].

Another category of pharmacological agents useful for vascular remodeling are represented by statins (3-hydroxy-3-methylglutaryl coenzyme A reductase inhibitors). Statins are primarily used to reduce cholesterol levels and the progression of atherosclerosis. However, their beneficial pleiotropic anti-inflammatory effects are equally well known and established. As it is well known, the treatment with pravastatin has been associated with reduced metalloproteinases and macrophage infiltration and cardiac expression of TGF Beta [52]. Studies conducted on a Marfan mice model demonstrates a potential therapeutic effect of statins in the slowing of aortic root dilation and elastin loss, especially when added to conventional therapies such as losartan [53].

## 6. Surgery in HTADs

The surgical management of HTADs for pediatric patients is not infrequent. A growing experience in the last two decades has demonstrated encouraging results of surgery of the thoracic aorta in children [54,55]. The improvement in earlier diagnosis and the observation of this subgroup of patients with an abnormal enlargement of the thoracic aorta compared to the normal population has pushed cardiologists and cardiac surgeons to adopt a lower threshold for aortic dimension in order to prevent aortic dissection or aortic catastrophic events. Much larger aortic diameters (more than 50 mm) regardless of age have been observed in this population and described by several authors [56].

At the same time, there have been no specific values for the pediatric population, and some authors argued that surgical management may be considered when the aortic diameter is twice the reference diameter [57]. Patel and colleagues suggested an aortic root diameter of 4 cm or a 0.5 cm/year progression of aortic dilatation as the cut-off for aortic root replacement in children with LDS [54]. These patients were particularly prone to developing aortic dissection at an early age. Everitt and colleagues reported 36% of aortic dissection as an indication for surgery on the thoracic aorta in LDS [58].

All the thoracic aorta “in toto” from the aortic root to the aortic arch, as well as the thoracic descending aorta, may be involved in the dilation process. Generally, the surgical techniques used to manage HTAD are well established and derived from adult experience. The Bentall–De Bono operation, described in 1968, was the treatment of choice for several years to replace the aortic valve and ascending aorta in patients with HTAD [54]. The technique consists of the implantation of a composite valved tube graft with coronary reimplantation. Despite it no longer being used as the first technique of choice, it remains the most frequently adopted solution when the aortic valve cannot be saved or when aortic dissection is the first presentation. The limitations of this type of surgery may be, for some authors, the need for an adequate size of the composite tube related to the growth of the children, but this problem may be overcome considering that children with HTAD generally have aortic root dimensions larger than normal that permits the implantation of an adult-sized prosthesis. Carrel and colleagues observed a mean preoperative aortic diameter of 41 mm in a series of 26 young patients with a mean age at operation of 10 years, whereas Everitt described a 47 mm mean aortic diameter and a mean implanted graft dimension of 25.6 mm [57,58].

In recent years, however, comparing the limitations associated with the use of prosthetic materials versus the physiologic performance of the native aortic valve, valve-sparing aortic root repair (VSARR) has been largely adopted in the pediatric population with HTAD. In a large series of 100 VSARR, 90% of patients operated were children with MS or LDS [55]. Aortic root remodeling and aortic root reimplantation were the most frequently used techniques, despite some authors describing the use of Florida sleeves as well. These techniques consist of replacing the aortic root with a Dacron tube graft and preserving the native aortic valve. The main difference between the first two techniques is that reimplantation permits the fixing of the aortic annulus instead of remodeling, preventing further dilatation. Both techniques have shown optimal results in terms of survival, with up to 95% at ten years in some series, but reimplantation (David V with Valsalva graft) seems to be associated with greater freedom to reoperate with respect to remodeling, and probably, as demonstrated by the results for the adult population, this is due to the lack of annular stabilization with the remodeling technique. Moreover, other advantages of VSRR lie in the mitigation of important drawbacks associated with prosthetic replacement, such as the necessity of life-long anticoagulation. These operations may be attractive for the pediatric population, where adherence to medical therapy may sometimes be difficult.

On the contrary, the necessity of reoperation may sometimes influence the parents to opt for a Bentall operation in order to avoid further procedures for the child.

Surgery for BAV includes the management of ascending aorta aneurysms in almost 50% of patients. Current guidelines recommend ascending aorta replacement in cases of a diameter > 45 mm if aortic valve surgery (repair or replacement) is planned. Ascending aorta replacement, even if mildly dilated, has been associated with the durability of BAV repair. The Ross operation in this population has shown excellent mid- to long-term results. Ivanov and colleagues reported an early mortality of 1.3% and a freedom from reintervention of 90% at 10 years. The Ross operation and VSRR carry the advantage in this period of life regarding the growth of the autograft and for the avoidance of anticoagulation [38,59]. Criticisms of the Ross operation have been that this operation transforms a one-valve disease into a two-valve disease, with drawbacks related to the pulmonary valve. Therefore, several authors suggest postponing the Ross operation beyond infancy, ideally into adulthood, and recommend native aortic valve preservation in growing children [60].

Considering the involvement of the descending thoracic aorta in patients with HTAD, it is worth mentioning the increasing use of thoracic endovascular aortic repair (TEVAR). TEVAR has become an attractive alternative to the conventional open surgical approach. Considered an off-label procedure offered especially to high risk patients, the results were not encouraging at the beginning, with up to 25% of recurrence of aortic dissection and mortality rate [61,62]. Moreover, the studies published were all virtually conducted on an adult population with a median age between 45 and 50 years. The data published in the last five years reported that between 18% and 44% of patients underwent TEVAR requiring conventional surgery, and the rate of the recurrence of the endoleak was 27% [61,62,63]. The most encouraging results were recently published by Kato and colleagues especially in terms of the mortality rate, which was reported to be 5% [63]. However, acute type B aortic dissection was described as one of the major complications of this subgroup of patients. For these reason, considering the magnitude of a conventional open approach management of the descending thoracic aorta in HATD, the possibility of using a TEVAR in this subgroup of patients may be evaluated in order to find the procedure with the best risk-adjusted ratio for these “fragile” patients.

## 7. Pregnancy in HTADs

HTADs are a large spectrum of diseases and pregnant women may be at risk of aortic dissection, especially women with MS, LDS, or vEDS [5,64,65].

In a Dutch national study [66], aortic dissection represented one of the main cardiovascular causes of maternal death; most dissections occurred in the third trimester (40%) and postpartum (35%). In some cases, BAV syndrome may be an increased risk when associated with marked dilation of the ascending aorta; pregnancy should be considered safe if the aorta size is <45 mm [67].

No maternal deaths were reported in a recent series from the multicenter registry ROPAC, but 2% of women presented an acute aortic dissection during pregnancy [68].

According to the European guidelines [69], pregnancy should be discouraged in women with MS when the aortic diameter exceeds 45 mm. However, the aortic dimension is not the sole parameter to be taken into account. Other parameters such as a family history of dissection, the progression of aortic dilation, and gene mutation may affect prognosis.

Women with LDS can be affected by a high risk of pregnancy-related complications, such as aortic dissection and uterine rupture, but recent data documented a lower risk of complications [70].

In women with vEDS, pregnancy is contraindicated even in the absence of aortic dilation; 12% of pregnancy-related mortality is reported to be due to arterial or uterine rupture [71]. The risk of dissection seems as high as 100-fold in women with TS. Pregnancies are rare because of frequent infertility but may sometimes occur in women with mosaic TS or in women who underwent assisted reproductive therapy. Measuring the aortic size corrected for body surface area is recommended, because women with TS have a short stature. Pregnancy should be contraindicated for women with TS and with an aortic size index ≥25 mm/m^2^ or ≥20–25 mm/m^2^ and other risk factors (hypertension, BAV or elongation of the transverse aorta) [69].

Prophylactic surgery should be considered when aortic dilation exceeds thresholds defined for different conditions [1,5].

All women with HTAD should undergo pre-conceptional counselling from adolescence about the risks of aortic dissection related to pregnancy, and appropriate aortic imaging with transthoracic echocardiography, cardiac magnetic resonance or computed tomography is recommended [5]. During pregnancy, careful management with periodic cardiologic consultations to assess the aortic diameter is recommended [5,64]. Strict control of blood pressure is advised to prevent values exceeding 130/80 mmHg. BBs are the drug of choice for the treatment of systemic hypertension, while ARBs are contraindicated in pregnancy due to fetal toxicity [64].

For most cases, vaginal delivery with epidural pain relief is the first choice, except for women with an aorta >45 mm in which an elective caesarean section is preferable [64,69].

The progression of the aortic diameter after pregnancy is not completely defined and requires a regular follow-up after delivery and at 4–8 weeks later [64].

## 8. Sport Activity in HTADs

The knowledge about the impact of physical exercise on aortic dilation is very limited. No randomized trials have ever been conducted on patients with aortopathies practicing competitive sports; hence, there is still a gap in scientific evidence [72].

Aortic dilatation is not a typical feature of athletes, being present in 0.03% [73].

Physical exercise can theoretically cause parietal stress, particularly power sports, producing a blood pressure overload in the aorta and determining an increased risk of aortic events. In a case series of 49 patients with aortic dissection during sports activities, 42 had a type A dissection, and most were associated with weightlifting [72].

Hemodynamic, genetic, and classic cardiovascular risk factors (i.e., age, sex, and smoke) are the most important determinants of aortic dilatation. Patients with aortic disease are predisposed to dilation and events at a younger age, therefore requiring regular follow-ups depending on the risk [74].

The European guidelines propose a risk stratification and subsequent indications of physical exercise depending on three factors [74]: valve morphology (BAV or tricuspid valve), aortic dimension and etiology of aortic disease. Generally, these patients have a higher risk of sudden death or dissection and aortic rupture as aortic size increases, but in some cases, such as EDS, the event can also occur in the case of normal diameters. Before starting physical activity, patients with aortopathy should undergo further evaluations with advanced imaging such as CMR or CCT and an exercise test. Competitive sports are recommended only for low-risk individuals [74]. Generally, power exercises are not recommended, while skill sports with a lower impact on blood pressure are preferred [75].

According to the ESC guidelines [74], patients with MS are classified as low to intermediate risk. Patients with BAV have limitations on sports activity related to the severity of the valve disease and the size of the aorta. Most of the studies conducted on BAV patients have not shown correlations between the intensity of sports activity and aortic dilatation over time [76].

There is no strong evidence to discourage moderate physical exercise in individuals with aortopathy. Risk stratification is crucial because of the need to identify patients with a progression of aortic dilation and at increased risk of fatal events over time [74,75].

## 9. Conclusions

In conclusion, patients with HTAD represent a heterogeneous population in which an accurate multimodality-integrated imaging approach is required for diagnosis and the subsequent follow-up. However, the evaluation of the etiology, which often requires genetic analyses, is fundamental to define clinical recommendations (for example, counselling for sports activity or pregnancy) and, when necessary, medical therapies and surgical management.

Dedicated services should be considered in order to provide an optimal multidisciplinary approach. Multicenter studies are needed, especially for rare conditions, to improve knowledge and the quality of care.

## Figures and Tables

**Table 1:** Clinical and morphologic features in different HTADs. BAV = bicuspid aortic valve; AC = aortic coarctation.

Aortopathy	Clinical Red Flags	Aortic Segment Involved
Marfan	Family history Mitral prolapse Dural ectasia Lens ectopia Iridodesis Skeletal abnormalities Pneumothorax–pulmonary emphysema	Sinus Valsalva
Ehlers–Danlos syndrome type IV	Fragile, translucent, premature aging of the skin Characteristic facial appearance Hyperextensibility of joint Extensive bruising	Thoracic and abdominal aorta
Loeys–Dietz syndrome	Hypertelorism Bifid uvula Craniofacial features (cleft palate, proptosis, etc.)	Distal to the aortic root
Turner syndrome	Short stature, webbed neck, broad chest, obesity Congenital lymphedema Ovarian failure/infertility Metabolic and hormonal alterations (hyperlipidemia, impaired glucose tolerance, diabetes) Congenital heart disease (BAV, AC)	- Transvers arch - Ascending aorta
BAV	Family history Aortic regurgitation/stenosis Congenital heart disease (AC)	- Sinus Valsalva - Tubular ascending aorta - Combined.

**Table 2:** Additional genes associated with familial thoracic aneurysm and rare syndromes. * The paucity of publications relating to variants in these genes means there is not sufficient evidence to support a definitive association with HTAD. LDS = Loyes–Dietz Syndrome; MS = Marfan Syndrome.

Gene Symbol	Disease	OMIM
*EFEMP2*	Cutix laxa, autosomal recessive, type 1B	604633
*ELN*	Cutix laxa, autosomal dominant	123700
*FBN2*	Congenital contractural arachnodactyly	121050
*FLNA*	Periventricular nodular heterotopia	300049
*NOTCH1*	Bicuspid aortic valve with aneurysm	109730
*SLC2A10*	Arterial tortuosity syndrome	208050
*SMAD4*	Juvenile polyposis/hereditary hemorrhagic telangiectasia syndrome	175050
*SKI*	Shprintzen–Goldberg syndrome	182212
*BGN* *	LDS-like and MS-like	301870
*FOXE3* *	Aortic aneurysm, familial thoracic 11	617349
*HCN4* *	Sick sinus syndrome 2	163800
*MAT2A* *	Aortic dilatation, bicuspid aortic valve	-
*MFAP5* *	Aortic aneurysm, familial thoracic 9	616166
*TGFB3/SMAD2* *	LDS type 5 and 6	615582, unassigned

## Data Availability

No new data were created or analyzed in this study. Data sharing is not applicable to this article.

## Supplementary Materials

The following supporting information can be downloaded at https://www.mdpi.com/article/10.3390/diagnostics14010112/s1. Table S1: Medical treatment and doses in HTADs [41,45,77,78,79,80,81,82,83,84].
