# Supplementary material for: Hereditary Thoracic Aortic Diseases
Source: Diagnostics (Basel). 2024 Jan 4;14(1):112. doi: 10.3390/diagnostics14010112 (PMC10795846; doi:10.3390/diagnostics14010112)
Supplement: Supplementary file 1 [file diagnostics-14-00112-s001.zip › diagnostics-2663280-supplementary.pdf]

Table S1: Medical treatment and doses in HTADs (order by year of publication).

Author	Type of aorthopathy	Design	Primary outcome	Age (mean)	Medical treatment/comparison	Mean dose	Ref
Shores, J. et al.	MFS	Open label randomized trial	ARD and clinical event	14.5-15.4	Propranolol vs placebo	212 +/- 68 mg given, four divided doses per day	1
Yetman et al.	MFS	Prospective, non-randomized, open-label	ARD rate of change per year; Aortic stiffness index and distensibility	12.8 ± 7.8	Propranolol/ atenolol/ enalapril	Propranolol: 1 mg/kg twice a day<br>Atenolol: 25 mg twice a day<br>Enalapril: 5 mg twice a day	2
Selamet, T. et al.	MFS	Observational, Retrospective	Rates of change in ARD. Cardiac complications (death, AD or CS)	9	Atenolol vs propranolol	0.92 mg/kg/day for propranolol<br>Metoprolol: dose not reported	3
Ong KT	vEDS	Multicenter, randomized, open trial	Arterial events (rupture or dissection, fatal or not)	36	Celiprolol vs placebo	Dosage error in the original article.	4
						100 mg celiprolol, titrated to 200 mg, 300 mg, and 400 mg per day	4
Chiu HH	MFS	Randomized, open-label, active-controlled trial	ARD	13.1± 6.3	Losartan vs BB (atenolol or propranolol)	Losartan: start 0.7 mg/kg daily → 50 mg/day or the maximum tolerable dose<br>BB: 50 mg of atenolol/day; propranolol twice daily for adults and 1 mg/kg/day for children	5
Sandor, G.G. et al.	MFS and LSD	Clinical trial randomized, double-blind study	Vascular function	17	Atenolol vs losartan	Atenolol: 25–50 mg<br>Losartan 25 mg daily	6
Mullen, M. et al.	MFS	Placebo-controlled, double-blind andomized trial	ARD	18	Irbesartan vs placebo	75 mg for all, and after randomization, 300 mg once daily (as tolerated) vs placebo	7
Frank et al.	vEDS	Retrospective cohort study	vEDS-related events and deaths	34,5	Celiprolol vs usual care	≤400 mg/day	8
Van Driest et al.	Variants in ADRB1 and CYP2C9 genes in MFS	Randomized trial	Annual rate of change in the maximum ARD (z-score)	11.5	Atenolol vs losartan	Atenolol: maximum tolerate dose. Daily dose max 250 mg<br>Losartan: 1.4mg/kg (maximum daily dose 100 mg)	9
Olfe, J. et al.	Genetic aortopathies	Retrospective study	Aortic root dimension	8-10	Predominantly metropolol vs valsartan	Metropolol:1–2 mg/kg bw/day.<br>Valsartan:1-3mg/kg bw/day	10

Table Legend: MFS, Marfan's syndrome; vEDS, vascular Ehlers–Danlos syndrome; LDS Loeys–Dietz syndrome; AD, aortic dissection; CS, cardiac surgery; CV, cardiovascular; ARD, aortic root dimension; BB, b-blockade; NA not available.

References table

1. Shores J, Berger KR, Murphy EA, Pyeritz RE. Progression of aortic dilatation and the benefit of long-term beta-adrenergic blockade in Marfan's syndrome. N Engl J Med. 1994 May 12;330(19):1335-41. doi: 10.1056/NEJM199405123301902. PMID: 8152445.
2. Yetman AT, Bornemeier RA, McCrindle BW. Usefulness of enalapril versus propranolol or atenolol for prevention of aortic dilation in patients with the Marfan syndrome. Am J Cardiol. 2005 May 1;95(9):1125-7. doi: 10.1016/j.amjcard.2005.01.032. PMID: 15842990.
3. Selamet Tierney ES, Feingold B, Printz BF, Park SC, Graham D, Kleinman CS, Mahnke CB, Timchak DM, Neches WH, Gersony WM. Beta-blocker therapy does not alter the rate of aortic root dilation in pediatric patients with Marfan syndrome. J Pediatr. 2007 Jan;150(1):77-82. doi: 10.1016/j.jpeds.2006.09.003. PMID: 17188619.
4. Ong KT, Perdu J, De Backer J, Bozec E, Collignon P, Emmerich J, Fauret AL, Fiessinger JN, Germain DP, Georgesco G, Hulot JS, De Paepe A, Plauchu H, Jeunemaitre X, Laurent S, Boutouyrie P. Effect of celiprolol on prevention of cardiovascular events in vascular Ehlers-Danlos syndrome: a prospective randomised, open, blinded-endpoints trial. Lancet. 2010 Oct 30;376(9751):1476-84. doi: 10.1016/S0140-6736(10)60960-9. Epub 2010 Sep 7. Erratum in: Lancet. 2016 Aug 6;388(10044):564. Dosage error in published abstract; MEDLINE/PubMed abstract corrected; Dosage error in article text. PMID: 20825986.
5. Chiu HH, Wu MH, Wang JK, Lu CW, Chiu SN, Chen CA, Lin MT, Hu FC. Losartan added to β-blockade therapy for aortic root dilation in Marfan syndrome: a randomized, open-label pilot study. Mayo Clin Proc. 2013 Mar;88(3):271-6. doi: 10.1016/j.mayocp.2012.11.005. Epub 2013 Jan 12. PMID: 23321647.
6. Sandor GG, Alghamdi MH, Raffin LA, Potts MT, Williams LD, Potts JE, Kiess M, van Breemen C. A randomized, double blind pilot study to assess the effects of losartan vs. atenolol on the biophysical properties of the aorta in patients with Marfan and Loeys-Dietz syndromes. Int J Cardiol. 2015 Jan 20;179:470-5. doi: 10.1016/j.ijcard.2014.11.082. Epub 2014 Nov 8. PMID: 25465809.
7. Mullen M, Jin XY, Child A, Stuart AG, Dodd M, Aragon-Martin JA, Gaze D, Kiotsekoglou A, Yuan L, Hu J, Foley C, Van Dyck L, Knight R, Clayton T, Swan L, Thomson JDR, Erdem G, Crossman D, Flather M; AIMS Investigators. Irbesartan in Marfan syndrome (AIMS): a double-blind, placebo-controlled randomised trial. Lancet. 2019 Dec 21;394(10216):2263-2270. doi: 10.1016/S0140-6736(19)32518-8. Epub 2019 Dec 10. PMID: 31836196; PMCID: PMC6934233.
8. Frank M, Adham S, Seigle S, Legrand A, Mirault T, Henneton P, Albuisson J, Denarié N, Mazzella JM, Mousseaux E, Messas E, Boutouyrie P, Jeunemaitre X. Vascular Ehlers-Danlos Syndrome: Long-Term Observational Study. J Am Coll Cardiol. 2019 Apr 23;73(15):1948-1957. doi: 10.1016/j.jacc.2019.01.058. PMID: 30999998.
9. Van Driest SL, Sleeper LA, Gelb BD, Morris SA, Dietz HC, Forbus GA, Goldmuntz E, Hoskoppal A, James J, Lee TM, Levine JC, Li JS, Loeys BL, Markham LW, Meester JAN, Mital S, Mosley JD, Olson AK, Renard M, Shaffer CM, Sharkey A, Young L, Lacro RV, Roden DM. Variants in ADRB1 and CYP2C9: Association with Response to Atenolol and Losartan in Marfan Syndrome. J Pediatr. 2020 Jul;222:213-220.e5. doi: 10.1016/j.jpeds.2020.03.064. PMID: 32586526; PMCID: PMC7323908.
10. Olfe J, Kanitz JJ, Stark VC, Stute F, von Kodolitsch Y, Biermann D, Huebler M, Kozlik-Feldmann R, Mir TS. Prophylactic effect of angiotensin receptor blockers in children with genetic aortopathies: the early bird catches the worm. Clin Res Cardiol. 2023 May 9. doi: 10.1007/s00392-023-02221-4. Epub ahead of print. PMID: 37160466.
